# Novel Antiretroviral Structures from Marine Organisms

**DOI:** 10.3390/molecules24193486

**Published:** 2019-09-26

**Authors:** Karlo Wittine, Lara Saftić, Željka Peršurić, Sandra Kraljević Pavelić

**Affiliations:** University of Rijeka, Department of Biotechnology, Centre for high-throughput technologies, Radmile Matejčić 2, 51000 Rijeka, Croatia

**Keywords:** antiretroviral agents, anti-HIV, marine metabolites, natural products, drug development

## Abstract

In spite of significant advancements and success in antiretroviral therapies directed against HIV infection, there is no cure for HIV, which scan persist in a human body in its latent form and become reactivated under favorable conditions. Therefore, novel antiretroviral drugs with different modes of actions are still a major focus for researchers. In particular, novel lead structures are being sought from natural sources. So far, a number of compounds from marine organisms have been identified as promising therapeutics for HIV infection. Therefore, in this paper, we provide an overview of marine natural products that were first identified in the period between 2013 and 2018 that could be potentially used, or further optimized, as novel antiretroviral agents. This pipeline includes the systematization of antiretroviral activities for several categories of marine structures including chitosan and its derivatives, sulfated polysaccharides, lectins, bromotyrosine derivatives, peptides, alkaloids, diterpenes, phlorotannins, and xanthones as well as adjuvants to the HAART therapy such as fish oil. We critically discuss the structures and activities of the most promising new marine anti-HIV compounds.

## 1. Introduction

Human immunodeficiency virus (HIV) infections pose a global challenge given that in 2017, according to the World Health Organization data, 36.9 million people were living with HIV and additional 1.8 million people were becoming newly infected globally (Table 1)**.** HIV targets immune cells and impairs the human defense against pneumonia, tuberculosis, and shingles as well as certain types of cancer [1]. The most advanced stage of HIV infection is the Acquired Immunodeficiency Syndrome (AIDS), which can take from two to 15 years to develop, depending on the individual [2].

**Figure 1:**
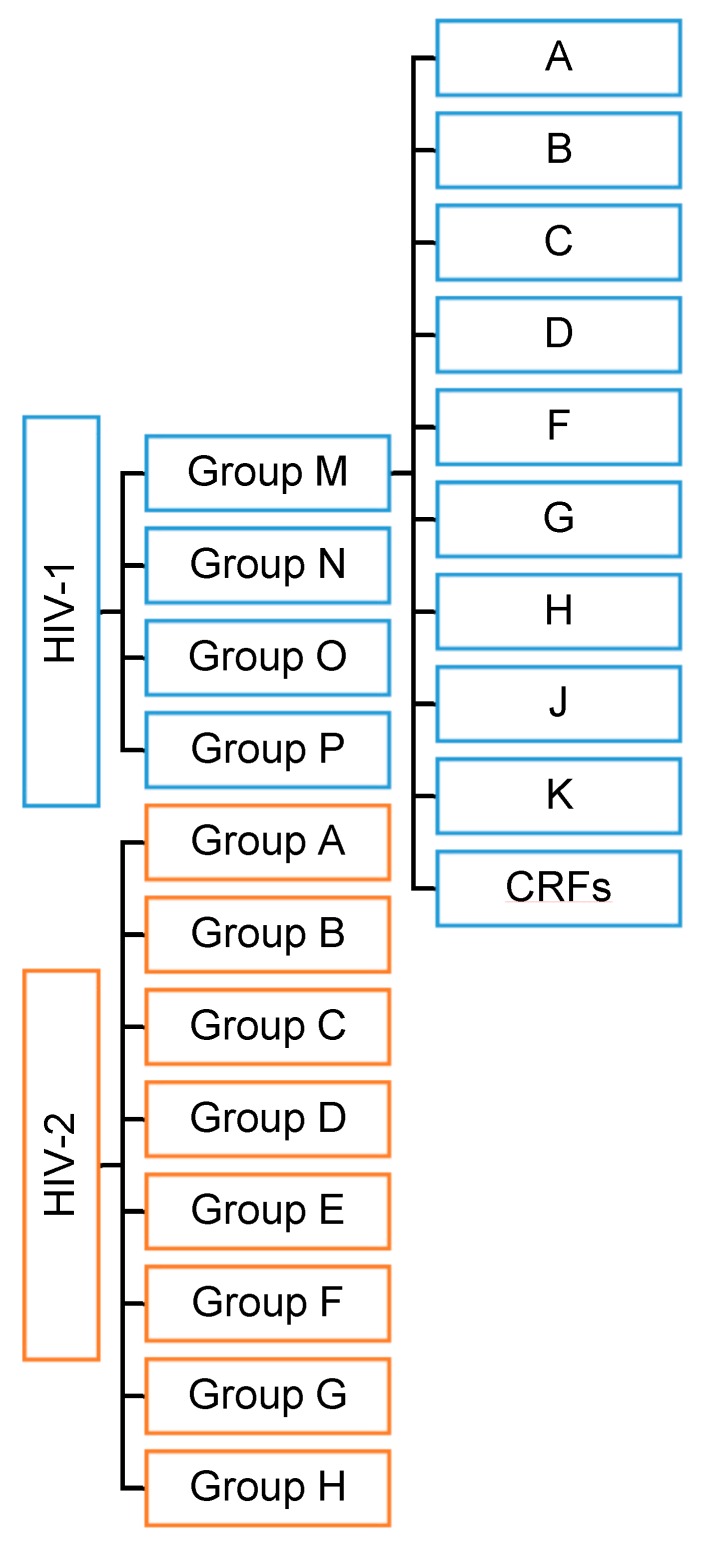
HIV types and strains classification.

HIV has two viral forms: HIV-1 (the most common form that accounts for around 95% of all infections worldwide) and HIV-2 (relatively uncommon and less infectious). HIV-1 consists of groups M, N, O, and P with at least nine genetically distinct subtypes of HIV-1 within group M (A, B, C, D, F, G, H, J, and K). Additionally, different subtypes can combine genetic material to form a hybrid virus known as the ‘circulating recombinant form’ (CRFs) (Figure 1). HIV-2 consists of eight known groups (A to H). Of these, only groups A and B are pandemic. The HIV-2 mechanism is not clearly defined and neither is its difference from HIV-1. However, the transmission rate is much lower in HIV-2 than in HIV-1. HIV-2 is estimated to be more than 55% genetically distinct from HIV-1.

The HIV-1 genome has reading frames coding for structural and regulatory proteins. The *gag* gene encodes the Pr55Gag precursor of inner structural proteins p24 (capsid protein, CA), p17 (matrix protein, MA), p7 (nucleoprotein, NC), and p6 involved in the virus particle release. The *pol* gene encodes the Pr160GagPol precursor of the viral enzymes p10 (protease, PR), p51 (reverse transcriptase, RT), p15 (RNase H), and p32 (integrase, IN). The *env* gene encodes the PrGp160 precursor of the gp120 (surface glycoprotein, SU) and gp41 (transmembrane protein, TM). Other genes include *tat,* encoding p14 (transactivator protein), *rev,* encoding p19 (RNA splicing regulator), *nef,* encoding p27 (negative regulating factor), *vif,* encoding p23 (viral infectivity protein), *vpr,* encoding p15 (virus protein r), *vpu,* encoding p16 (virus protein unique), *vpx* in HIV2, encoding p15 (virus protein x), and *tev,* encoding p26 (tat/rev protein) [3].

The HIV infections are extremely problematic as the virus targets the CD4+ memory T-cells population, which is essential for organism immunity. HIV can attach itself to the host cell through 1) a relatively nonspecific interaction with negatively charged cell-surface heparan sulfate proteoglycans [4], 2) specific interactions between the Env and α4β7 integrin [5,6], and/or 3) the interaction with pattern-recognition receptors, such as the dendritic cell-specific intercellular adhesion molecular 3-grabbing non-integrin (DC-SIGN) [7]. The attachment of HIV in any of the abovementioned ways can increase the efficacy of infection because it brings Env, a heavily glycosylated trimer of gp120 and gp41 heterodimers, into close proximity with the viral receptor CD4 and co-receptor [8]. Finally, in order for the viral entry to occur, Env needs to bind itself to the host protein CD4 [9,10].

The binding of the HIV glycoprotein gp120 to the host cell CD4 receptor causes conformational changes of the gp120 glycoprotein, which uncover additional binding sites that interact with distinct proteins on the host cell membrane, known as β-chemokine co-receptors (mainly CCR5 and CXCR4), which facilitate the virus entry into the cell [11].

After the infection, a progressive decline of CD4 + cells consequently leads to the failure of the immune system function and the development of opportunistic infections that usually lead to death [1]. In HIV-infected patients, immunodeficiency develops both as a result of the viral replication and the failure of the patients’ homeostatic mechanisms. The continuous viral presence in the patients after the application of therapy is attributed to the CD4 + T-cell homeostasis owing to a pool of latently infected and resting CD4 + T-cells, macrophages, and follicular dendritic cells that remain in the organism. Indeed, the complex interactions of the patient’s immune system with the virus, and vice versa after the viral suppression, are thought to be crucial for the control of disease progression [12,13]. 

Current therapeutic approaches mainly target proteins that are vital for the viral cycle. One of the prominent examples is the linear 36-amino acid synthetic peptide enfuviritide (T20, Fuzeon), developed by Hoffmann-La Roche, and the first FDA approved fusion inhibitor for the treatment of HIV-1/AIDS acting through the binding to the gp41 subunit of the HIV-1 envelope glycoprotein. This induces a conformational change that brings the viral and cellular membranes into close enough proximity for the fusion and the subsequent viral entry into the host-cell to occur. Nevertheless, several restrictions, such as a low genetic barrier for drug resistance and a short in vivo half-life, limit its clinical use [14,15,16,17].

Other various FDA-approved antiretroviral drugs from seven mechanistic classes of inhibitors of the HIV replication are also available for the treatment of infected patients, namely, the nucleoside reverse-transcriptase inhibitors NRTIs, non-nucleoside reverse-transcriptase (RT) inhibitors NNRTIs, protein inhibitors PIs, fusion inhibitors, entry inhibitors—CCR5 co-receptor antagonists, HIV integrase strand transfer inhibitors, and multi-class combinations. None of the mentioned drug classes alone or in combination, the latter being known as the highly active antiretroviral therapy (HAART), can eradicate the HIV infection, and effective vaccines remain unavailable.

The difficulties of HIV-1 vaccine research are, in part, a result of 1) the unavailability of a model for natural immunity related to HIV; 2) the existence of genetically distinct subtypes of HIV and frequent mutations; 3) unidentified correlates of specific immune response to HIV; 4) lack of a reliable, non-human animal model for HIV infection (SIV in monkeys vs. HIV in humans).

The established latent pro-viral reservoirs in the patient’s body can stochastically begin to reproduce viral particles, which makes the HIV disease practically incurable. From over 160 compounds identified so far as latency-reversing agents (LRAs), none have led to a promising cure [18].

Several rare and long-term remissions of HIV cases are described in the literature. For example, Berlin, London, and Düsseldorf’s patients underwent bone marrow transplantation with stem cells from a donor with a rare genetic mutation of the CCR5. The Mississippi baby received a very early antiretroviral therapy that extended the time of the viral rebound for more than 27 months. There undoubtedly remains a lot to be learned from these cases, and further investigation of stem-cell transplantation in people living with HIV is required [19,20,21]. 

The currently used antiretroviral treatment, alone or in combination, extends the quality and life expectancy of HIV-infected individuals but does not cure them. Drug resistance, along with the emergence of drug-resistant virus strains, a high-cost of the lifetime treatment regimen, cell toxicity, and serious side effects of currently used anti-HIV drugs [2] underlie the need for a synthetic development of new drugs or the search for active anti-HIV molecules in natural sources. Mother Nature has been perfecting its chemistry for three billion years, and most of it has been done in water. Intense competition and feeding pressure as well as non-static marine environmental conditions yield compounds with chemical and structural features generally not found in terrestrial natural products. 

New efficient molecules directed against HIV should demonstrate better performance in comparison with the currently approved drugs and suppress the HIV virus and/or eliminate the latent HIV reservoirs present in the human body. 

Around 60% of drugs currently available on the market are derived or inspired by nature [22]. Turning to nature for drug development holds great potential, especially when it comes to marine organisms. Only few marine-derived drugs have been approved on the market so far but many are in the preclinical or clinical stage of development [23]. Marine organisms make up to two-thirds of Earth’s species and produce, as a consequence of living in a highly competitive environment, unique and structurally diverse metabolites. Over the last 40 years, bioprospecting efforts have resulted in over 20,000 compounds of marine origin. The highest share of marine metabolites (up to 70%) are obtained from marine sponges, corals, and microorganisms, while mollusks, ascidians, and algae metabolites form only a minor part [24]. Oceans are, indeed, still a rather underexploited habitat, and biodiversity appears to be higher in the oceans than on land, which might be relevant when focusing on the marine environment as an untapped reservoir of novel antiretroviral candidates. In the discovery of new antiviral marine-derived drugs, researchers usually implement two strategies. They either screen the extracts from different strains (e.g., cyanobacteria, microalgae) or search directly for bioactive molecules in organisms—extract and purify them for evaluation within the drug development pipeline. It is thought that the marine environment might yield more potent anti-HIV candidates characterized by a higher efficiency (lower effective dose) and a better selectivity and which do not induce resistance development. This could, of course, only be speculation based on some of the previous success stories in the discovery of drugs from natural sources such as, e.g., lovastatin and paclitaxel. However, nature generally does create more sophisticated and perfected systems with a complex mode of action. 

An excellent example is protein lectin, derived from marine red algae *Griffithsia* sp. named Griffithsin with mid-picomolar activities, which groups it among the most potent HIV entry inhibitors reported so far [25]. It inhibits the HIV infection by binding itself to high mannose glycan structures on the surface of gp120, altering the gp120 structure or its oligomeric state [26]. This interaction relies on the specific trimeric “sugar tower,” including N295 and N448 [27]. Griffitshin can also prevent infections caused by other glycoprotein-enveloped viruses such as the Ebola virus, hepatitis C virus, and the severe acute respiratory syndrome coronavirus. It has been shown that the dimerization of Griffithsin is necessary for a high potency inhibition of HIV-1 [28]. However, the discrepancy between the HIV gp120 binding activity and the HIV inhibitory activity points to the presence of mechanism unrelated to a merely simple HIV gp120 binding [26]. The most promising application of Griffithsin would be its incorporation into vaginal and rectal gels, creams, or suppositories acting as an antiviral microbicide to prevent the transmission of HIV.

Despite the vast number of structurally diverse and unique bioactive molecules from the marine environment, the global marine pharmaceutical pipeline includes only eight approved drugs: Adcetris^®^, Cytosar-U^®^, Halaven^®^, Yondelis^®^, Carragelose^®^, Vira-A^®^, Lovaza^®^, and Prialt^®^ [29]. Overall, it has taken 20 to 30 years from their discovery to their entry into the market. A sustainable supply, structural complexity, optimization of formulation, and ADMET properties, and a scale-up issue have prevented further development of several highly promising marine compounds. It is by no means an easy task to identify a marine candidate that may be considered as a potential drug. Initial high costs of developing a natural product into a drug could be balanced out with careful long-term considerations (biodiversity, supply, and technical, market) [30]. 

This paper provides an overview of natural marine metabolites that were first identified in the period between 2013 and 2018 or the previously identified marine constituents with a recently confirmed anti-HIV activity that could be potentially used or further optimized as novel ant-HIV agents. We also comprehensively summarize anti-HIV activities for several categories of marine structures including chitosan and its derivatives, sulfated polysaccharides, lectins, bromotyrosine derivatives, peptides, alkaloids, diterpenes, phlorotannins, and xanthones as well as fish oil as an auxiliary to HAART therapy.

## 2. Marine Compounds in the Treatment of HIV/AIDS

### 2.1. Chitosan and Its Derivatives

**Figure 2:**
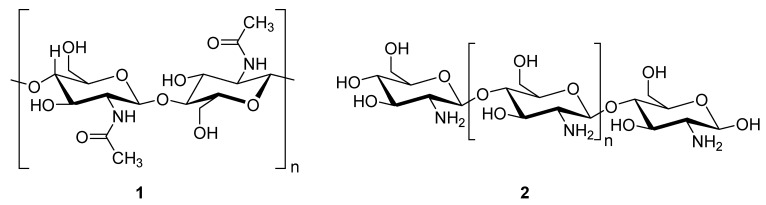
Chemical structures of chitin (**1**) and chitosan (**2**).

Chitosan (**2**, Figure 2), a natural marine byproduct, is a poly-cationic linear polysaccharide derived from chitin (**1**, Figure 2) after partial deacetylation. Chitin is a structural element in the exoskeleton of mainly shrimps and crabs and is mainly composed of the randomly distributed *β*-(1-4)-linked D-glucosamine and *N*-acetyl-D-glucosamine. It has been previously shown that this compound can exhibit a large scale of different bioactivities and can also be used as a carrier for anti-HIV drugs [31]. Chitosan is loaded with saquinavir, an anti-HIV drug with a protease inhibitory activity, which showed better cell targeting efficiency than saquinavir alone [32]. Furthermore, trimethyl chitosan has improved Atripla, an anti-HIV drug consisting of efavirenz, emtricitabine, and tenofovir disoproxil fumarate, anti-HIV 1 activity, and has allowed it to be used in lower concentrations [33]. The antiretroviral activity is manifested in the chitosan-specific cationic nature that allows the formation of electrostatic complexes or multilayer structures with other negatively charged polymers [34]. Karagozlu et al. reported about new QMW-COS and WMQ-COS oligomers with anti-HIV activities. These oligomers are conjugates of chitosan and the Gln-Met-Trp peptide, which were constructed as a continuation of the authors’ previous research, in which a high potency of synthetically constructed chitosan oligomers was confirmed in anti-HIV therapy. More specifically, it was shown that these oligomers suppress syncytium formation, which occurs as a fusion of infected cells with neighboring cells, induced by HIV in a dose-dependent manner. However, the authors also noticed that after a certain period, the number of syncytia once again increased, suggesting that the cells should be re-treated with QMW-COS and WMQ-COS oligomers to maintain the primary therapeutically-relevant effect. The inhibition of the HIV-1 induced lytic effect, determined by the cell viability assay, showed that IC_50_ for QMW-COS was 48.14 µg/mL and was almost identical for WMQ-COS, 48.01 µg/mL. These oligomers effectively reduced the HIV load but showed no effects on HIV-1 RT and protease in vitro. Higher dosages were also required for the reduction in the HIV-1IIIB p24 antigen production assessed by the ELISA assay and the HIV-1_RTMDR_ p24 antigen production. The highest difference between the compounds was reflected in IC_50_ values obtained from studies on the virus-induced luciferase activity in infected cells, where QMW-COS had a higher potency in comparison with WMQ-COS. Lastly, the authors determined the effects of oligomers on the interaction between gp41 and CD4 by using the CD4-gp41 ELISA assay, whereby both oligomers showed high potency. The effect of these oligomers was highest when they were applied immediately upon the HIV-1 infection of cells, indicating that they should be used as a potential treatment in the early stages of HIV infection, probably at the entry stage [31]. 

### 2.2. Sulfated Polysaccharides

Sulfated polysaccharides (SP) are the most studied class of antiviral polysaccharides that are structural components of the alga cell wall where they play both the storage and structural role. They are an important source of galactans, commercially known as agar and carrageenan in red alga (Rhodophyta), fucans (fucoidan, sargassan, ascophyllan, and glucuronoxylofucan) in brown alga (Phaeophyta), and ulvans-sulfated heteropolysaccharides that contain galactose, xylose, arabinose, mannose, glucuronic acid, or glucose [35,36,37]. Many studies indicate that, in marine algae, sulfated polysaccharides facilitate water and ion retention in extracellular matrices, which is an important mechanism for coping with desiccation and osmotic stress in a highly salted environment [38,39,40]. The antiviral activity of this group of compounds is mainly connected to the degree of sulfation, constituent sugars, molecular weight, conformation, and dynamic stereochemistry [41,42]. The effect of counter cation should also be considered as an important factor in observed biological activity.

The antagonizing effect of the negatively charged sulfated polysaccharides on the HIV-1 entry into cells may be due to 1) their binding onto the positively charged V3 domain of gp120, thereby preventing the virus attachment to the cell surface [43,44,45] or 2) the masking of the docking sites of gp120 for sCD4 on the surface of T lymphocytes, thereby disrupting the CD4-gp120 interaction [46,47,48] and subsequently inhibiting the expression of the viral antigen and the activity of the viral reverse transcriptase [49,50].

#### 2.2.1. Heparan Sulfate

**Figure 3:**
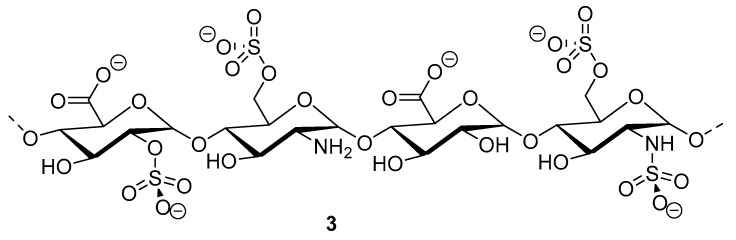
Structure of heparan sulfate (**3**).

Heparinoid polysaccharides can interact with the positive-charge regions of cell-surface glycoproteins, leading to a shielding effect on these regions, which prevents the binding of viruses to the cell surface [51]. The sulfated polysaccharides content in marine mollusks is high in comparison with the bovine mucosal heparin (73.5%) and the porcine mucosal heparin (72.8%) [52]. The acidic sulfate groups on heparin (**3**, Figure 3), or heparin-like compounds, can inhibit HIV through electrostatic interactions with basic amino-acid residues of the transcriptional activator Tat protein [53].

#### 2.2.2. Fucose Containing SP

So far, the main anti-infectious activities documented for the fucose-containing SP are those against viruses [54]. More importantly, these polysaccharides are selective inhibitors of various enveloped viruses, including HIV [54,55,56]. FCSP acts during the early phase of infection by blocking the virus attachment and entry into the host cells, but may also inhibit subsequent replication stages in vitro [57].

#### 2.2.3. Fucoidans

Three fucoidans extracted from three brown seaweeds (*Sargassum mcclurei*, *Sargassum polycystum*, *Turbinara ornata*) inhibit the early stages of HIV-1 entry into target cells, with IC_50_ ranging from 0.33 to 0.7 µM. Neither the sulfate content nor the position of sulfate groups are related to the anti-HIV activity of fucoidans, suggesting the involvement of other structural parameters such as the molecular weight, the type of glycosidic linkage, or even a unique fucoidan sequence [56]. Although the presence of sulfo-groups seems to be necessary for anti-HIV activity [58], these data do not support random sulfation as the main antiviral factor.

**Figure 4:**
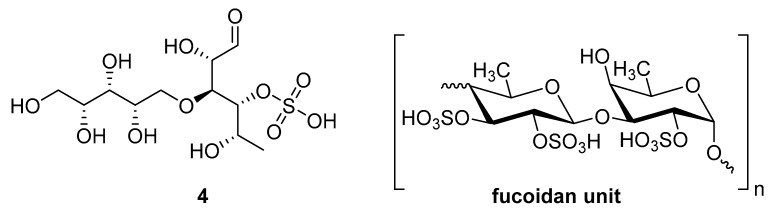
Structure of ascophyllan (**4**) and fucoidan unit.

Sulfated fucan polysaccharides, ascophyllan (**4**, Figure 4)**,** and two fucoidans (S and A) (**5** and **6**, Table 2), derived from different sources, significantly inhibit (IC_50_ 1.3; 0.3; 0.6 µg/mL) the early step of HIV-1 (R9 and JR-Fl) infection. They also inhibit the VSV-G-pseudotype HIV-1 infection in HeLa cells [59].

**Figure 5:**
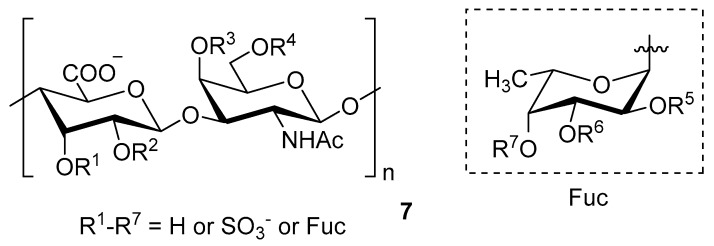
General chemical structure of fucosylated chondroitin sulfate (**7**).

Chondroitin sulfate with fucosylated branches (FuCS) (**7**, Figure 5) has also attracted attention as an HIV antiviral compound. Depolymerized fucosylated CS, extracted from the sea cucumber, has shown in vitro activity against a range of viral strains, including the resistant ones [60]. FuCS is effective in blocking the laboratory strain HIV-1IIIB entry and replication by inhibiting the p24 antigen production (4.26 and 0.73 μg/mL, respectively) and the infection of the clinic isolate HIV-1KM018 and HIV-1TC-2 (23.75 and 31.86 μg/mL, respectively) as well as suppressing the HIV-1 drug-resistant virus. Additionally, FuCS is also effective in T-20-resistant strains (EC50 values ranging from 0.76 to 1.13 μg/mL). The depolymerized fragments seem to maintain a similar anti-HIV action at the early stages of infection, apparently through interaction with an HIV envelope glycoprotein gp120. The sulfated fucose branches appear necessary for antiviral activity, which is also affected by molecular weight and carboxylation [61]. While the in vitro results of the fucosylated CS against HIV are promising, it is questionable whether the antiviral activity would be maintained in vivo. Other polyanionic HIV entry inhibitors, which advanced into clinical trials, failed to prove effective against the heterosexual HIV-1 transmission. This was related to factors not considered in previous development stages, such as the presence of seminal plasma and the concentration and retention of polyanionic inhibitors [62].

The complex chemical architecture and the sulfate patterning of marine polysaccharides depends on numerous factors (species, tidal cycles, environmental variations (e.g., salinity), harvesting season, plant age, geographical location etc.) [39,63,64,65,66,67,68,69], making isolation, purification, and comprehensive chemical characterization a highly challenging task [70]. The development of many polysaccharides into clinical application is hindered by the still limited view of their sophisticated and diverse nature. Despite having good antiviral effects, the use of carbohydrate drugs is still in its infancy, and intensive structure-activity and in vivo studies are needed in the future. 

A relatively new strategy in inducing immunity and developing an HIV vaccine is to use carbohydrates. The major difficulty of such an approach lies in mimicking the specific glycan protective epitope. Gp120 of HIV is a highly glycosylated envelope surface glycoprotein responsible for the receptor and co-receptor binding, which, together with gp41, comprises the heterodimeric envelope trimer spikes of HIV. *N*-linked glycans, mainly mannose and complex-type, cover much of the gp120 surface-accessible face of the HIV envelope spike forming the glycan shield. Inadequate mimicry of the glycan shield, tolerance mechanisms, and/or the inability to induce a domain-exchange are reflecting difficulties in creating the proper specificity of Abs [71]. Most of the vaccines for HIV-1 in preclinical trials are based on a Manα1-2Man oligomannosyl epitope (various conjugates, engineered yeast strains, and modified glycoproteins) [72,73,74,75,76,77,78,79]. Better specificity could potentially be gained using carbohydrates of marine origin. 

### 2.3. Lectins

Lectins are a group of proteins that specifically, but reversibly, bind glycosylated molecules on the cell surface. Precisely, this group of molecules can affect cell-cell interactions, protect cells from pathogens, influence cell adhesion, and affect the intracellular glycoprotein translocation [80]. Recently, lectins have become promising agents for antiretroviral therapy, and different researches have confirmed their anti-HIV properties. Their antiretroviral activity is manifested through an alteration of the interaction between HIV gp120 or gp41 and the corresponding receptors [81], which, in the end, inhibit the HIV cell function, HIV infectivity, and the formation of the syncytium, multi-nucleated cells [82,83,84]. 

Several published review papers describe the previously found marine lectins with antiretroviral action [85,86]. For example, Gogineni et al. reported about some new, unusual lectins, such as the *β*-galactose specific lectin (CVL), CGL, DTL, DTL-A, SVL-1, and SVL-2 [86]. Additionally, Akkouh et al. reported about some new algal lectins, such as *Boodlea Coacta* Lectin, Griffithsin and *Oscillatoria Agardhii* Agglutinin (OAA), and some cyanobacterial lectins, such as Cyanovirin-N, Scytovirin, Microcystis Viridis Lectin, and Microvirin. 

However, in the last few years, there has not been as much research focused on anti-HIV lectins from marine sources. Only Hirayama et al. (2016) reported about the new high-mannose specific lectin and its recombinants that possess anti-HIV activity [87]. In their research performed on the red alga *Kappaphycus alvarezii,* authors confirmed KAA-1 and KAA-2, two KAA mannose-binding lectin isomers, as potent anti-HIV agents. The anti-HIV role of action of these two compounds includes a strong binding to the virus envelope glycoprotein gp120 and, consequently, the inhibition of HIV entry into the host cells. These KAA recombinants, as well as the native one, inhibited the HIV-1 entry at IC_50_s (neutralization assay in Jurkat cells) of 7.3–12.9 nM. Authors concluded in the end that KAAs, besides their strong inhibitory effect on HIV entry into the cells, have a potential as agents in treatments against other viruses possessing high mannose glycans on their envelope as well. 

### 2.4. Peptides

It has been shown that the majority of marine peptides have strong anti-HIV activity. They are usually isolated from marine organisms through the process of enzymatic hydrolysis [88]. The most common source of such constituents is marine sponges that are known for their unique metabolome [89] and are a source of more than 36% of all marine bioactive compounds [90]. Their bioactive peptides can be found in cyclic or linear forms and contain unusual amino acids that form unique structures rarely found in other species. Antiretroviral activity of such structures works on several different levels: blocking of virus entry, inhibition of the cytopathic viral activity, neutralization of viral particles, or inhibition of viral fusion and entry [89,91].

**Figure 6:**
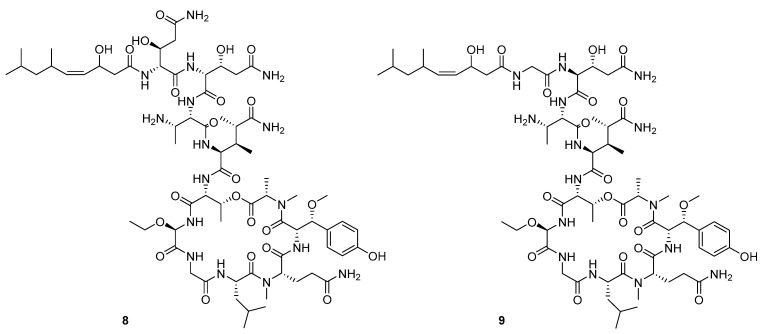
Structures of stelletapeptin (**8**) A and stelletapeptin B (**9**).

Recently, Shin et al. discovered two new depsipeptides from marine sponges *Stelletta sp*., stellettapeptin A (**8**, Figure 6), and stellettapeptin B (**9**, Figure 6), with the inhibition of the cytopathic effect of HIV-1 infection [92]. Confirming the mentioned theory about the unique metabolome of marine sponges, the authors revealed that these two compounds have previously undescribed nonproteinogenic amino-acid parts on peptides that are rarely found in nature. Namely, stellettapeptin A and stellettapeptin B have an unexpected polyketide subunit, 3-hydroxy-6,8-dimethylnon-4-enoic acid, 3-OHGln, and 3-OHAsn residues. Their high potency is witnessed through low EC_50_ values (inhibition of the cytotoxic effect upon HIV infection)—values of 23 nM for stellettapeptin A and 27 nM for stellettapeptin B.

**Figure 7:**
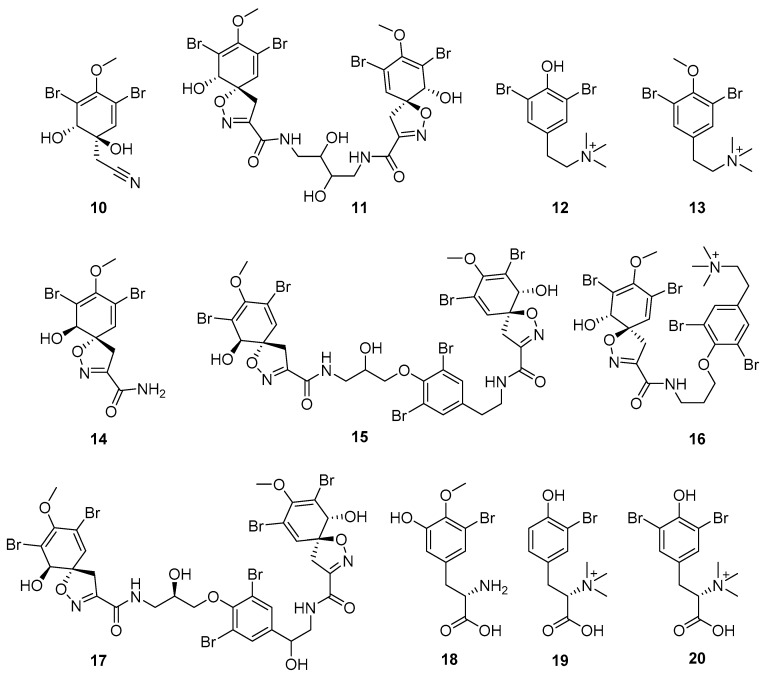
Structures of aeroplysinin-1 (**10**), dihydroxyaerothionin (**11**), 3,4-dibromo-*N,N,N*-trimethyltyraminium (**12**), 3,5-dibromo-*N,N,N,O*-tetramethyltyraminium (**13**), purealidin R (**14**), 19-deohxyfistularin 3 (**15**), purealidin B (**16**), fistularin-3 (**17**), 3-bromo-5-hydroxy-*O*-methyltyrosine (**18**), 3-bromo-*N,N,N*-trimethyltyrosinium (**19**), and 3,5-dibromo-*N,N,N*-trimethyltyrosinium (**20**).

Furthermore, newly discovered anti-HIV constituents derived from marine sponges *Verongula rigida* and *Aiolochoria crassa* with amino-acid structure were published by Gomez-Archila et al. (2014) [93]. In their paper, they evaluated and confirmed the anti-HIV effect of 11 bromotyrosine derivatives (Table 3), whereby aeroplysinin-1 (**10**), 19-deoxyfistularin 3 (**15**), purealidin B (**16**), fistularin 3 (**17**) and 3-bromo-5-hydroxy-O-methyltyrosine (**18**, Figure 7) were the most potent in their anti-HIV activity. Aeroplysinin 1 (**15**) and purealidin B (**16**), compounds found in *V. rigida* species inhibited the HIV-1 replication in a dose-dependent manner by more than 50%. Specifically, for aeroplysinin 1, HIV-a replication was inhibited by 74% at a concentration of 20 µM, whereas purealidin was less potent with inhibitory power of 57% at a concentration of 80 µM. These two compounds had been previously isolated; however, their anti-HIV activity was proven in this research. The same was with 3-bromo-5-hydroxy-*O*-methyltyrosine (**18**) that has a relatively high percentage of inhibition of HIV activity (47%) in a dose-dependent manner. However, the exact mechanism of action remains unclear. In the same study, additional tests with these compounds on the HIV RT inhibition (qPCR of the early and late transcripts), nuclear import (qPCR analysis of 2-LTR transcript), and HIV entry inhibition (viral infectivity assay) were performed. The results showed that aeroplysinin-1 (**10**), 19-deoxyfistularin 3 (**15**), purealidin B (**16**), fistularin 3 (**17**), and 3-bromo-5-hydroxy-*O*-methyltyrosine (**18**) influenced the nuclear import of the HIV virus with around or more than 50% of inhibition: aeroplysinin-1 (**10**) showed 67% of inhibition at 10 µM, 19-deoxyfistularin 3 62% inhibition at 20 µM, purealidin B 66% of inhibition at 20 µM, fistularin 3 47% of inhibition at 10 µM, and 3-bromo-5-hydroxy-*O*-methyltyrosine 73% of inhibition at 80 µM. Viral RT inhibition was not high for all compounds, whereby the highest results were around 50% of inhibition. For example, purealidin B had 58% of inhibition at 20 µM in the qPCR analysis of early transcripts. As for the HIV entry inhibition, all compounds were active in a dose-depended manner, with the highest results of inhibition obtained for 3,5-dibromo-*N*,*N*,*N*,*O*-tetramethyltyraminium (**13**), from 14% to 30%. Finally, the authors stressed the structural similarity of these compounds with the HIV integrase and protease inhibitors, suggesting that these compounds can have a broader mode of antiviral action.

**Figure 8:**
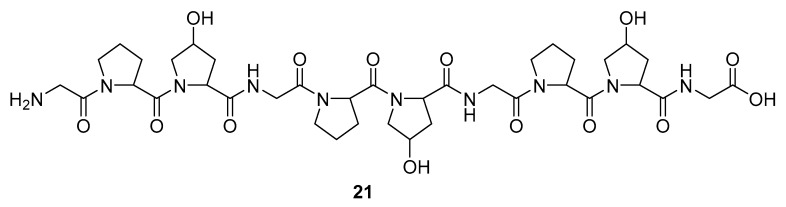
Structure of the Alaska Pollack collagen hydroxyl proline (APCHP) peptide (**21**).

Marine sponges are not the sole source of bioactive proteins. For example, Jang et al. reported about a new small hydroxyproline-rich peptide from Alaska Pollack collagen (APHCP, **21**, Figure 8) that exhibits a unique antiviral activity [94]. This peptide is a Gly-Pro-Hyp-Gly-Pro-Hyp-Gly-Pro-Hyp-Gly peptide, and the authors showed that the most important part of a peptide for anti-HIV activity is the hydroxyl group at hydroxyproline, whereas a peptide with only prolines does not exhibit antiviral activity. Its anti-HIV 1 mode of action is manifested through the inhibition of the induced syncytia formation by the interference of an HIV fusion, inhibition of cell lysis, RT activity, and the production of the p24 antigen. It was shown that APHCP can decrease the HIV-1 induced cell lysis at a potency around EC_50_ of 459 µM (EC_50_ against anti-HIV-1 induced cell lysis—MTT assay). Additionally, through the inhibition of the viral RT at EC_50_ at 374 µM, this peptide’s crucial role in the inhibition of the conversion of viral RNA to DNA was also confirmed. With EC_50_ of 405 µM, this compound effectively suppressed the p24 production in viral cells, as determined by the Western blot analysis. 

Similarly, one new anti-HIV peptide was isolated from *Spirulina maxima* (SM-peptide) [95]–the Leu-Asp-Ala-Val-Asn-Arg peptide, and the authors showed its HIV-1 infection inhibition in a human T cell line MT4. The peptide inhibited cell lysis, p24 antigen production, and HIV-1 RT. Specifically, IC_50_ (obtained by a cell viability assay) against an anti-HIV 1 infection was determined as 0.691 mM, the inhibition of the HIV-1-induced RT activation (RT assay kit) in MT4 cells was at a high 90% at a concentration of 1.093 mM, and the p24 production (p24 antigen production assay) was inhibited at 95% at a concentration of 1.093 mM.

### 2.5. Alkaloids

Marine organisms are well-established sources of natural alkaloids. Although the term ‘alkaloid’ seems puzzling and is prone to scientific controversy, alkaloids are generally defined as nitrogen-containing compounds derived from plants and animals. Relatively few alkaloids from marine sources have been found to possess antiretroviral properties and, so far, none have found their clinical use.

**Figure 9:**
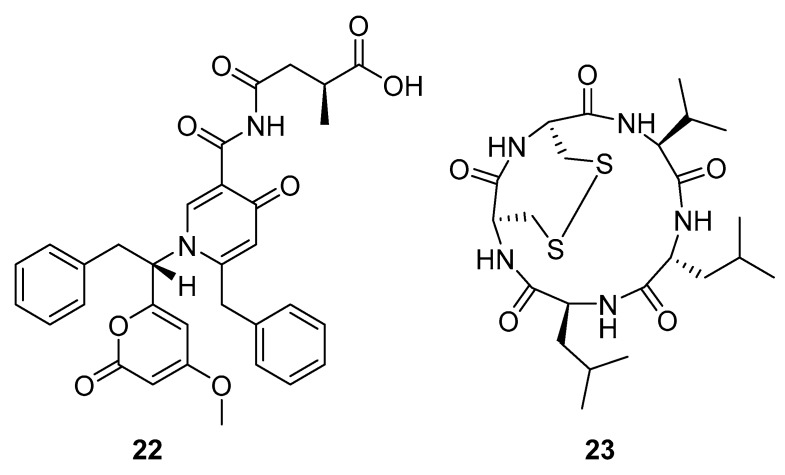
Structures of aspernigrin C (**22**) and malformin C (**23**).

Aspernigrin C (**22**, Figure 9) and malformin C (**23,** Figure 9) have been isolated from marine-derived black aspergili, *Aspergillus niger* SCSIO Jcw6F30, and their inhibitory activity against the chemokine receptor subtype 5 (CCR5) tropic HIV-1 SF162 has been evaluated. They show potent inhibition of infection with IC_50_ values 4.7 ± 0.4 µM and 1.4 ± 0.06 µM, which is comparable to the nucleoside reverse transcriptase inhibitor—abacavir (IC_50_ = 0.8 ± 0.1 µM) and the HIV-1 entry inhibitor ADS-J1 (IC_50_ = 0.8 ± 0.1 µM). In comparison to other aspernigrins, it has been suggested that the 2-methylsuccinic moiety is responsible for the potency of aspernigrin C [96].

**Figure 10:**
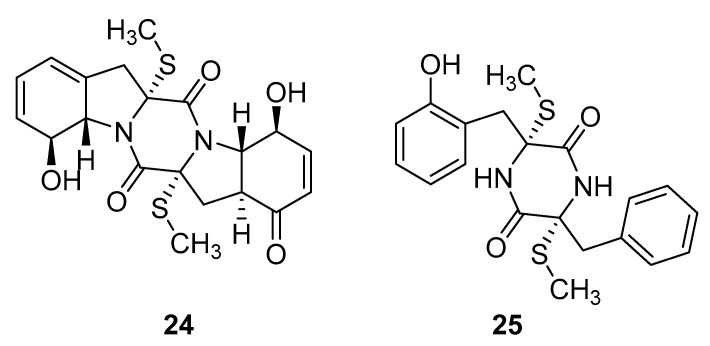
Structures of eutypellazine E (**24**) and eutypellazine J (**25**).

Thiodiketopiperazine-type alkaloids, eutypellazines A-M, isolated from the EtOAc extract of the fermentation broth of deep-sea sediment fungus *Eutypella sp*. shows potent inhibitory effects against pNL4.3.Env-.Luc co-transfected 293T HIV model cells. Eutypellazine E (**24**, Figure 10) exerts activity in a low micromolar range (IC_50_ = 3.2 ± 0.4 µM), while eutypellazine J (**25**, Figure 10) shows a reactivating effect toward latent HIV-1 in J-Lat A2 cells. This could be used as a promising strategy to expunge the HIV-1 infection by activating latent virus cellular reservoirs in combination with HAART [97].

**Figure 11:**
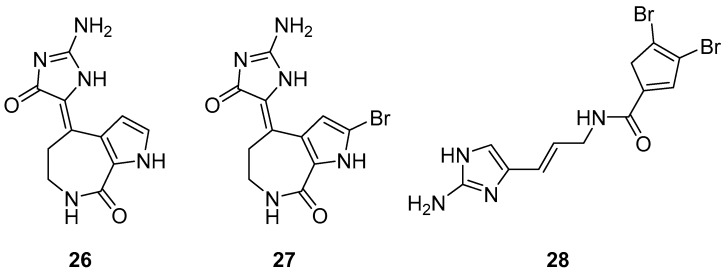
Structures of debromohymenialdisine (**26**), 10Z-hymenialdisine (**27**), and oroidin (**28**).

The *S. carteri* Red Sea sponge extract yields three previously characterized compounds: debromohymenialdisine (DBH) (**26**, Figure 11), hymenialdisine (HD) (**27**, Figure 11), and oroidin (**28**, Figure 11). DBH and HD exhibited a 30–40% inhibition of HIV-1 at 3.1 µM and 13 µM but with associated cytotoxicity. Conversely, oroidin displayed a 50% inhibition of viral replication at 50 µM without observed cytotoxicity. Also, it showed inhibition of HIV-1 reverse transcriptase up to 90% at 25 µMc [98].

**Figure 12:**
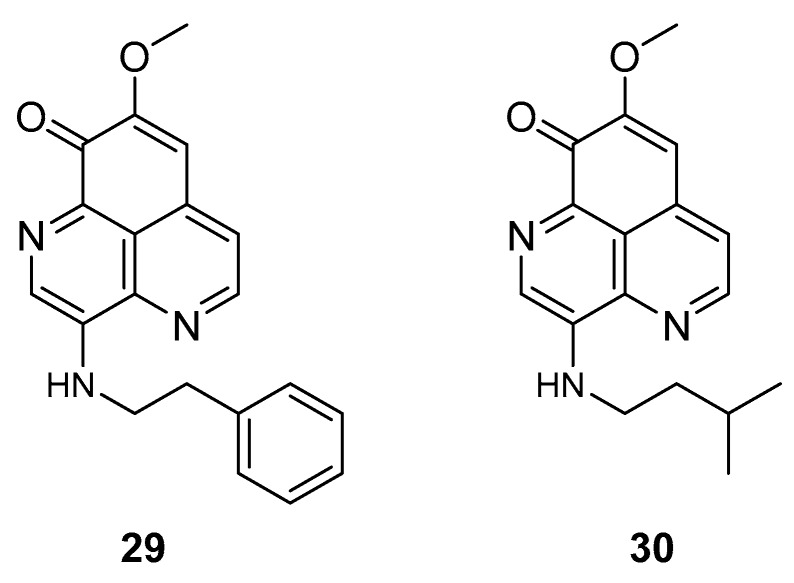
Structures of 3-(phenetylamino)dimethyl(oxo)aaptamine (**29**) and 3-(isopentylamino)dimethyl (oxo)aaptamine (**30**).

The two known alkaloids of the aaptamine family containing 1*H*-benz[*de*]-1,6-naphthyridine skeleton, namely 3-(phenetylamino)demethyl(oxy)aaptamine (**29**, Figure 12) and 3-(isopentylamino)demethyl(oxy)aaptamine (**30**, Figure 11), were isolated from the sponge *A. aptos*. They exhibited anti-HIV activity, with inhibitory rates of 88.0% and 72.3%, respectively, at a concentration of 10 µM [99].

**Figure 13:**
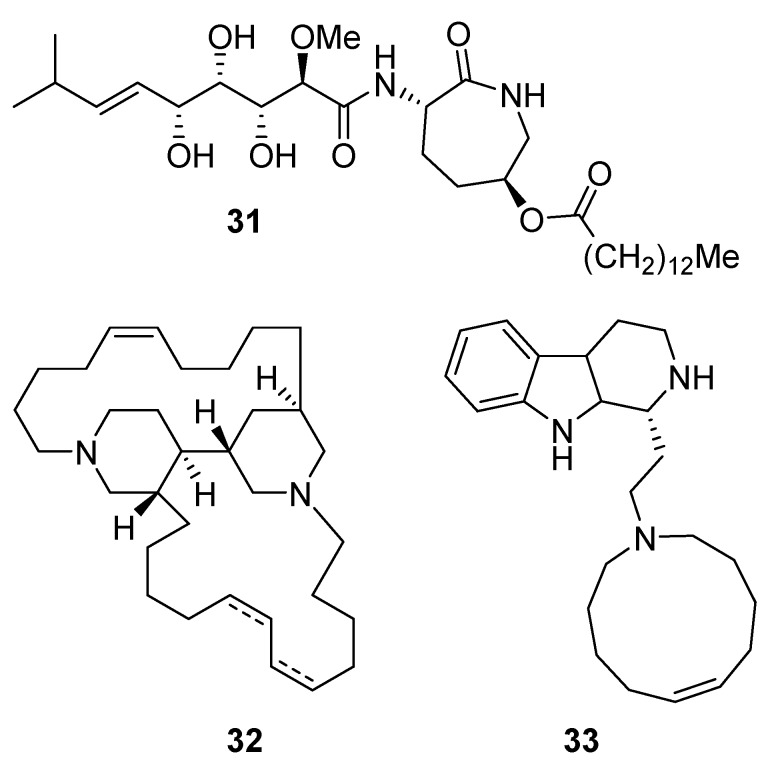
Structures of bengamide A (**31**), haliclonacyclamines A + B (**32**), keramamine C (**33**).

Bengamide A (**31**, Figure 13), haliclonycyclamine A+B (**32**, Figure 13) and keramamine C (**33**, Figure 13) inhibit HIV-1 with a 50% effective concentration of 3.8 µM or less. The most potent among them, bengamide A, blocked HIV-1 in a T cell line with an EC_50_ of 0.015 µM (which was comparable to control antiretrovirals indinavir 0.029 µM, efavirenz 0.0024 µM, and raltegravir 0.011 µM) and in peripheral blood mononuclear cells with EC_50_ of 0.032 µM. It was concluded that HIV-1 LTR NF-κB response elements are required for a bengamide A-mediated inhibition of LTR-dependent gene expression [100].

**Figure 14:**
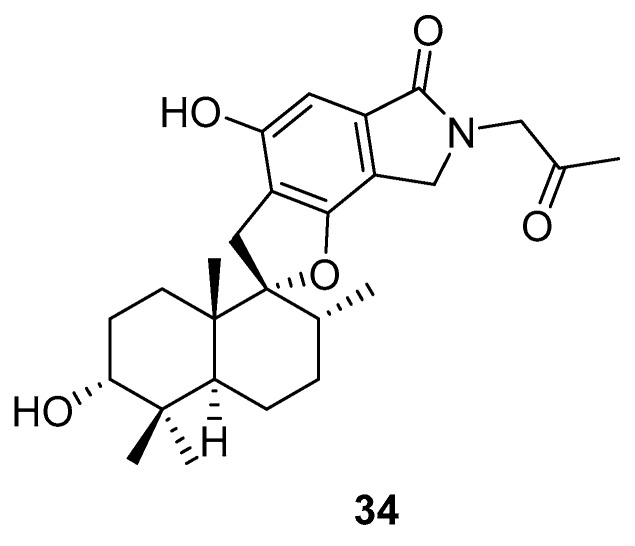
Structure of stachybotrin D (**34**).

Phenylspirodrimane, stachybotrin D (**34**, Figure 14) isolated from the sponge-derived fungus *Stachybotrys chartarum* MXH-X73, was discovered to be a HIV-1 *RT* inhibitor, which showed inhibitory effects on the wild type (EC_50_ 8.4 µM) and five NNRTI-resistant HIV-1 strains (EC_50_ 7.0; 23.8; 13.3; 14.2; 6.2 µM) [101].

### 2.6. Diterpenes

**Figure 15:**
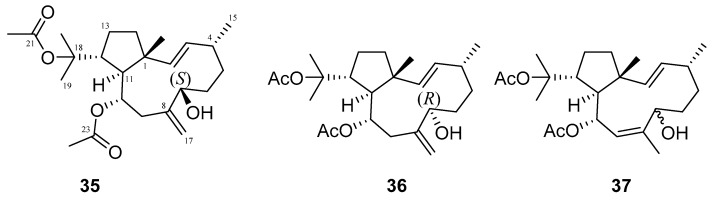
Structures of the new dolabellane diterpenoids dolabelladienols A–C (**35**–**37**).

Many terpenes from marine natural products demonstrated anti-HIV properties. Mechanisms of action involve blocking of different steps of the HIV-1 replicative cycle as reverse transcriptase inhibitors, protease inhibitors, or entry inhibitors. Among them, diterpenes from marine algae are nowadays in the spotlight due to their promising anti-HIV activities [102]. Dolabellane diterpenes are compounds from the diterpene group that have recently been extensively studied for their anti-HIV activity. Pardo-Vargas et al. characterized three new dolabellane diterpenes isolated from the marine brown alga *Dictyota pfaffii* from Northeast Brazil: (1R*,2E,4R*,7S,10S*,11S*,12R*)10,18-diacetoxy-7-hydroxy-2,8(17)-dolabelladiene, (1R*,2E,4R*,7R*,10S*,11S*,12R*)10,18-diacetoxy-7-hydroxy-2,8(17)-dolabelladiene, (1R*,2E,4R*,8E,10S*,11S,12R*)10,18-diacetoxy-7-hydroxy-2,8-dolabelladiene, named dolabelladienols A–C (**35**–**37**, Figure 15), respectively [102]. In particular, the new compounds, dolabelladienols A and B, showed potent anti-HIV-1 activities that can be confirmed with their low IC_50_ values of 2.9 and 4.1 μM and low cytotoxic activity against MT-2 lymphocyte tumor cells. These promising anti-HIV-1 agents were even more active than previously known 2,6-dolabelladienes series.

**Figure 16:**
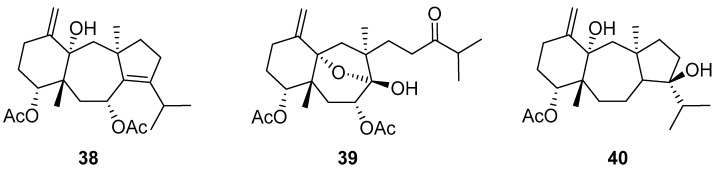
Structures of marine dolastanes (**38** and **40**) and secodolastane diterpene (**39**) derived from *Canistrocarpus cervicornis*.

De Souza Barros et al. tested marine dolastanes (**38**, **40**, Figure 16) and secodolastane diterpenes (**39**, Figure 16) isolated from the brown alga *Canistrocarpus cervicornis* for anti-HIV-1 activity [103]. They observed that the marine diterpenes **38**–**40** inhibit the HIV-1 replication in a dose-dependent manner (EC_50_ values of 0.35, 3.67, and 0.794 μM) without a cytotoxic effect (CC_50_ values ranging from 935 to 1910 μM). Additionally, they investigated the virucidal effect of these diterpenes and their potential use as microbicides. Dolastane-diterpenes **38** and **40** showed a potent effect on HIV-1 infectivity, whereas no virucidal effect was observed for secodolastane diterpene **39**, demonstrating another mechanism of antiretroviral activity. Therefore, the authors suggested a potential use of marine dolastanes **38** and **40** as microbicides that could directly inhibit virus infectivity and possibly act before the virus penetrates the target cells [103].

**Figure 17:**
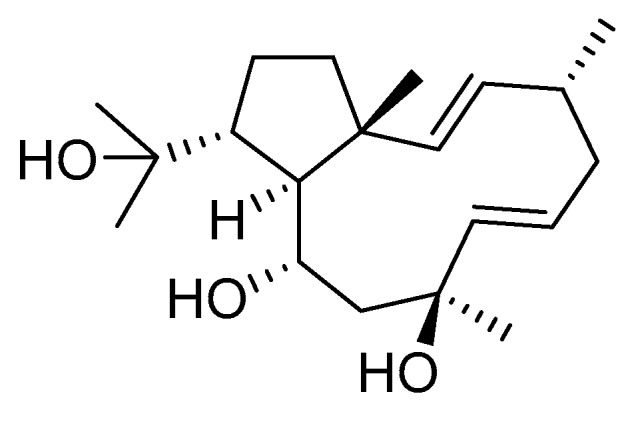
Structure of (1*R*,2*E*,4*R*,6*E*,8*S*,10*S*,11*S*,12*R*)-8,10,18-trihydroxy-2,6-dolabelladiene (**41**).

Dolabelladienetriol from brown alga *Dictyota* spp has also been evaluated as a potential microbicide against HIV-1 in tissue explants. Namely, Stephens et al. examined the 8,10,18-trihydroxy-2,6-dolabelladiene (**41**, Figure 17) in pretreated peripheral blood cells (PBMC) and macrophages along with their protective effect in the ex vivo explant model of the uterine cervix [104]. Pre-treatment of peripheral PBMC and macrophages with dolabelladienotriol showed inhibitory effects on HIV-1 replication. Furthermore, in the explant model dolabelladienetriol inhibited viral replication in a dose-dependent manner from 20 to 99% in concentrations of 0.15 and 14.4 μM without a loss in the viability of the tissue. The authors concluded that this compound has great potential as a possible microbicide. The same compound was also theoretically analyzed as an inhibitor of the wild-type and mutants’ HIV-1 reverse transcriptase [105]. Firstly, the structure-activity relationship studies revealed that a low dipole moment and high HOMO (highest occupied molecular orbital)-LUMO (lowest unoccupied molecular orbital) gap values are related to the antiviral activity. Secondly, molecular docking studies with RT wild-type and mutants showed a seahorse-like conformation of 8,10,18-trihydroxy-2,6-dolabelladiene, hydrophobic interactions, and hydrogen bonds with important residues of the binding pocket. Finally, the authors suggested a new derivative of the 8,10,18-trihydroxy-2,6-dolabelladiene with an aromatic moiety in the double bond to improve its biological activity.

**Figure 18:**
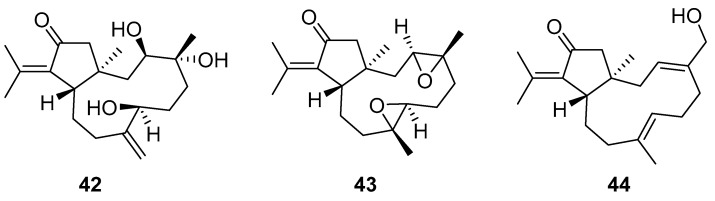
Structures of semi-synthesized oxygenated dolabellanes (**42**–**44**) originally isolated from the Caribbean octocoral *Eunicea laciniata*.

Although dolabellane diterpenes of brown alga *Dictyota* spp showed a strong anti-HIV-1 activity, this was not confirmed for dolabellane diterpenes isolated from octocorals. Therefore, some chemical transformations have been conducted to improve the anti-HIV-1 potency of the main dolabellane 13-keto-1(*R*),11(*S*)-dolabella-3(*E*),7(*E*),12(18)-triene from Caribbean octocoral *Eunicea laciniata* [106]. Oxygenated dolabellanes derivatives (**42**–**44**, Figure 18), obtained by epoxidation, epoxide opening, and allylic oxidation of ketodolbellatriene have shown significantly improved antiviral activities and a low cytotoxicity to MT-2 cells, which makes them promising antiviral compounds.

### 2.7. Phlorotannins and Xanthones

**Figure 19:**
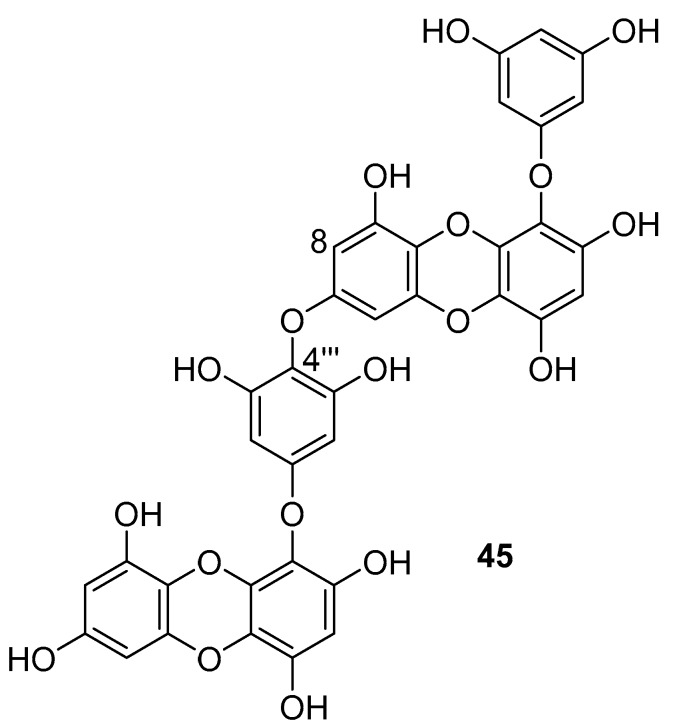
Chemical structure of 8,4′′′-dieckol (**45**) from *E. cava*.

Phlorotannins are tannin derivatives made from several phloroglucinol units linked to each other in different ways. Phlorotannins contain phenyl linkage (fucols), ether linkage (fuhalols and phlorethols), phenyl and ether linkage (fucophloroethols), and dibenzodioxin linkage (eckols) [86,107]. So far, a series of phlorotannins have been identified with potent anti-HIV activity. For example, 8,8′-bieckol and 6,6′-bieckol from marine brown alga *Ecklonia cava* has shown an enhanced HIV-1 inhibitory effect [112,113]. Karadeniz et al. reported that 8,4′′′-dieckol (**45**, Figure 19) is another phlorotannin derivative isolated from the same brown alga that could be used as a drug candidate for the development of new generation anti-HIV therapeutic agents [107]. The compound showed HIV-1 inhibitory activity at noncytotoxic concentrations. More precisely, the results indicated that 8,4**′′′**-dieckol inhibited the cytopathic effects of HIV-1, including HIV-1 induced syncytia formation, lytic effects, and viral p24 antigen production. Furthermore, 8,4**′′′**-dieckol inhibited an HIV-1 entry and RT enzyme with the inhibition ratio of 91% at a concentration of 50 μM.

**Figure 20:**
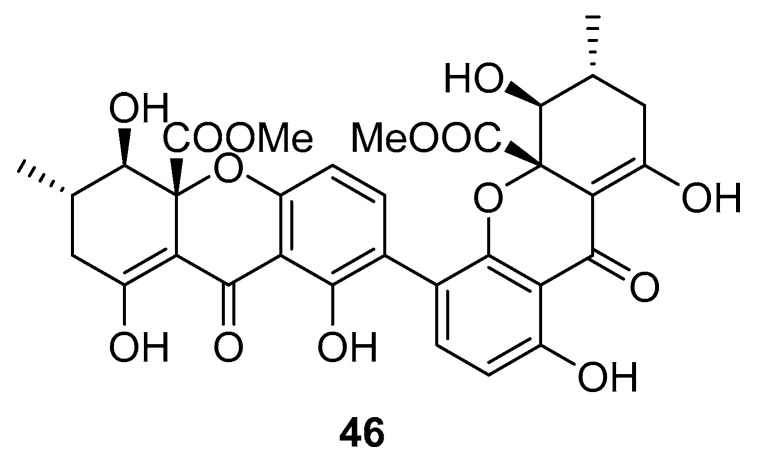
Structure of penicillixanthone A (**46**).

Recently, for the first time, xanthone dimer was identified as a potential anti-HIV-1 agent [108]. Xanthones are secondary metabolites from higher plant families, fungi, and lichen [114,115]. Although structurally related to flavonoids, xanthones are not as frequently encountered in nature [9]. Penicillixanthone A (PXA) (**46**, Figure 20), a natural xanthone dimer, has been isolated from the jellyfish-derived fungus *Aspergillus fumigates* with fourteen other natural products [108]. However, only penicillixanthone A showed inhibitory activities in an HIV infection. Marine-derived PXA displayed potent anti-HIV-1 activity against CCR5-tropic HIV-1 SF162 and CXCR4-tropic HIV-1 NL4-3, with IC_50_ of 0.36 and 0.26 μM, respectively. A molecular docking study confirmed that PXA might bind to either CCR5 or CXCR4 to prevent HIV entry into target cells. Therefore, PXA, as a CCR5/CXCR4 dual-coreceptor antagonist, may be seen as a new potential lead product type for the development of anti-HIV therapeutics.

### 2.8. Fish Oil as an Adjuvant to HAART Therapy

**Figure 21:**
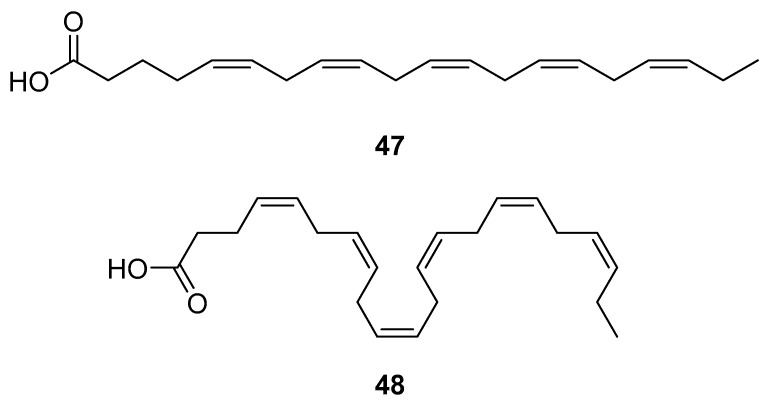
Structures of eicosapentanoic (**47**) and docosahexanoic acid (**48**).

HAART therapy can cause severe side effects, e.g., insulin resistance, lipoatrophy, dyslipidemia, and abnormalities of fat distribution. Therefore, finding an adequate diet and supplementation to lower the negative effects of the HAART combination therapy is desirable [116]. Fish oil contains omega-3 polyunsaturated fatty acids (PUFA), eicosapentaenoic (EPA, 20:5n-3) (**47**, Figure 21) and docosahexaenoic (DHA, 22:6n-3) (**48**, Figure 21) acids, which may have beneficial effects for HIV-infected patients. It has been shown that the addition of fish oil to the diet of HIV-infected individuals receiving usual antiretroviral therapy can significantly lower serum triglycerides levels [117], which is highly relevant knowing that HIV dyslipidemia is a serious problem related to an increased frequency of cardiovascular disease.

Recently, He et al. analyzed the influence of DHA on the locomotor activity in ethanol-treated HIV-1 transgenic rats [109]. The prevalence of alcohol use and alcohol abuse in infected individuals is much higher, and numerous ethanol and HIV-1 viral proteins have synergistic effects on inflammation in the central nervous system [118,119,120]. HIV remains in the body in its latent form after HAART therapy and, as such, can induce neuroinflammation. DHA depletion has been found to be associated with various neurological abnormalities, and its administration can have a neuroprotective effect. DHA taken daily could reverse the effects of the ethanol negative effect on the locomotor activity in the presence of HIV viral proteins. An in vivo study, using real-time quantitative PCR, showed that the addition of DHA can reduce elevated levels of IL-6, IL-18, and increase the expression of NF-κB in the striatum. This proved the potential of this fish oil constituent as an adjuvant in HIV patients’ treatment that can help in lowering the interactive effects of ethanol consumption during HIV infection.

### 2.9. Others

**Figure 22:**
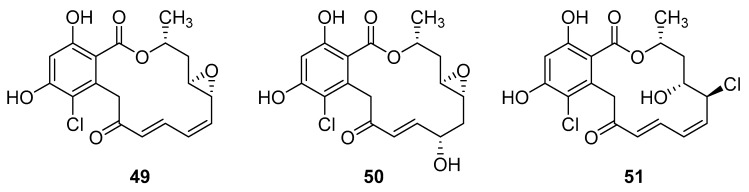
Structures of radicicol (**49**), pochonin B (**50**), pochonin C (**51**).

Resorcyclic acid lactones, namely radicicol (**49**, Figure 22) pochonin B (**50**, Figure 22) and C (**51**, Figure 22) isolated from *H. fuscoatra* exhibited a 92–98% reactivation efficiency of the latent HIV-1 relative to SAHA (subeoylanilide hydroxamic acid, vorinostat, HDAC inhibitor) and EC_50_ of 9.1, 39.6 and 6.3 µM [110]. The reactivation strategy is, indeed, a promising strategy to expunge the HIV-1 infection by reactivating latent viral loads, mainly in CD4 + T-cells, which quickly rebound when antiviral treatment is interrupted. It was noted that all active compounds contain Michael acceptor functionality. The PKC-independent mechanism of reactivation of the latent HIV-1 remains to be elucidated.

**Figure 23:**
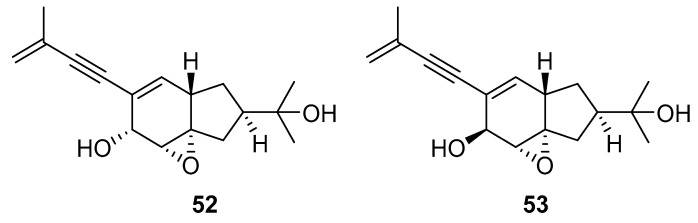
Structures of truncateols O (**52**) and P (**53**).

A team of researchers led by Zhao isolated new isoprenylated cyclohexanols from the sponge-associated fungus *Truncatella angustata* named truncateols O-V [111]. In vitro testing showed that truncateols O and P (**52** and **53**, Figure 23), analogues bearing the alkynyl group in the side chain, exhibit a significant inhibition toward the HIV-1 virus with IC_50_ values of 39.0 μM and 16.1 μM, respectively. These compounds could be considered as new anti-HIV lead compounds due to lower cytotoxicity (CC_50_ > 100 μM) in comparison with the positive control efavirenz (CC_50_ = 40.6 μM).

## 3. Future Directions in the Anti-HIV Marine Drug Development

Marine organisms have been acknowledged as a precious source of bioactive compounds that may provide novel anti-HIV structures or lead structures for structural optimization. A large amount of evidence from scientific research confirmed a high biological potential of these compounds to treat serious diseases, including infective ones. Some of the marine-derived bioactive compounds discovered much earlier have emerged with novel properties and potential applications after a decade or two. Isolation and structural elucidation of compounds from marine organisms is not an easy task and still carries challenges. Identification of all the compounds is a daunting task, especially with regards to complex structural motifs that may be present in a single marine extract. Taxonomic knowledge is still insufficient to enable unambiguous species classification that can result in the false prediction of chemical constituents and hamper structural analysis. Furthermore, a temporal lag between the discovery, chemical characterization, and associated pharmacological activities is quite common, and the majority of marine metabolites are usually tested for anticancer activity, whereas anti-HIV and other possible biological effects are neglected or mostly not performed due to a lack of funding. Targeted assays and in vivo analyses are similarly performed only for some of the potential candidates, while the translation into clinical trials remains very limited. Thus, the financial gap is certainly a relevant factor contributing to the slow drug development process in this area. In particular, the development of anti-HIV compounds, which act by mechanisms that differ from existing antivirals, requires a well-designed and focused approach to studying the mode of action. Libraries should be created for specifically defined crude extracts, their corresponding simplified fractions as well as for pure compounds for a well-balanced natural product discovery program. Additionally, there exist but few publications in which scientists have tried to modify known compounds of marine origin to improve their bioactivity. We are, however, continuously witnessing advancements in the deep-sea exploration technology, sampling strategies, genome sequencing, genome mining, genetic engineering, chemo-enzymatic synthesis, nanoscale NMR structure determination, and development and optimization of suitable fermentation strategies to ensure a continued supply of unique bioactive compounds from the oceans. Therefore, the grounds have been met for a broad, international effort based on scientific collaboration that would rely on well-equipped infrastructure and human resources as a prerequisite for a full advancement in the field and development of new drug candidates for the pharmaceutical market in the future.

## Figures and Tables

**Table 1:** Summary of the global human immunodeficiency virus (HIV) epidemic (2017) according to World Health Organization (WHO) data.

	People Living with HIV in 2017	People Newly Infected with HIV in 2017	HIV-Related Deaths in 2017
total	36.9 million (31.3–43.9 million)	1.8 million (1.4–2.4 million)	940,000 (670,000–1.3 million)
adults	35 million (29.6–41.7 million)	1.6 million (1.3–2.1 million)	830 000 (590,000–1.2 million)
women	18.2 million (15.6–21.4 million)		
men	16.8 million (13.9–20.4 million)		
children (<15 years)	1.8 million (1.3–2.4 million)	180,000 (110,000–260,000)	110,000 (63,000–160,000)

**Table 2:** Chemical composition of polysccharides (Fuc, Fucose; Xyl, Xylose; Glu, Glucose; Man, Mannose; Gal, Galactose) in ascophyllan, S- and A-fucoidan.

	Neutral Sugars						
	Fuc	Xyl	Glu	Man	Gal	Uronic acid	SO_3_^−^
ascophyllan (**4**)	15.5	13.4	0.3	3.4	0.6	21.4	9.6
S-fucoidan (**5**)	24.8	1.9	0.8	1	3.1	9.6	22.6
A-fucoidan (**6**)	28.4	4.3	2.0	0.8	5.1	5.8	19.4

**Table 3:** Summary of anti-HIV compounds from marine organisms.

Group	Compound	Location	Organism	Assay	Dose	Activity	Structure	Reference
Peptide + chitosan oligomer	QMW-COS	not disclosed ^a^	marine byproduct	IC_50_—inhibition of HIV-1 induced lytic effects (cell viability assay) IC_50_—inhibition of HIV-1_IIIB_ p24 antigen production (ELISA) IC_50_—inhibition of HIV-1_RTMDR_ p24 antigen production (ELISA) IC_50_—inhibition of virus-induced luciferase activity in infected TZM-bl cells IC_50_—inhibition of the interaction between gp41 and CD4 (CD4-gp41 ELISA)	48.14 µg/mL 67.35 µg/mL 81.03 µg/mL 68.13 µg/mL 39.13 µg/mL	**anti-HIV-1**; inhibition of the HIV entry at an early stage, blocking the fusion of HIV-1 infected cells, interference of gp41-CD4 binding	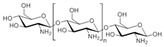 + glutamine (Q), methionine (M), tryptophan (W)	[31]
	WMQ-COS	not disclosed^a^	marine byproduct	IC_50_—Inhibition of HIV-1 induced lytic effects (cell viability assay) IC_50_—inhibition of HIV-1IIIB p24 antigen production (ELISA) IC_50_—inhibition of HIV-1_RTMDR_ p24 antigen production (ELISA) IC_50_—inhibition of virus-induced luciferase activity in infected TZM-bl cells IC_50_—inhibition of interaction between gp41 and CD4 (CD4-gp41 ELISA)	48.01 µg/mL 98.73 µg/mL 144.02 µg/mL 250 µg/mL 51.48 µg/ml	**anti-HIV-1**; inhibition of the HIV entry at an early stage	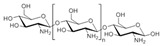 + tryptophan (W) methionine (M), glutamine (Q),	[31]
Sulfated polysaccharides	heparan sulfate (**3**)	not disclosed	-	EC_50_—inhibition of HIV-1_IIIB_ strain (syncytia assay) EC_50_—inhibition of HIV-1_IIIB_ strain (p24 assay) EC_50_—inhibition of HIV-1_IIIB_/H9 strain (co-cultivation assay) EC_50_—inhibition of HIV-1_RF_ strain (p24 assay) EC_50_—inhibition of HIV-1_KM018_ strain (p24 assay) EC_50_—inhibition of HIV-1_TC-2_ strain (p24 assay) EC_50_—inhibition of HIV-1_A17_ strain (p24 assay) EC_50_—inhibition of HIV-1_RF/V82F/184V_ strain (p24 assay) EC_50_—inhibition of HIV-1_L10R/M461/L63P/V82T/184V_ strain (p24 assay) EC_50_—inhibition of HIV-1_CBL-20_ strain (syncytia assay) EC_50_—inhibition of HIV-1_ROD_ strain (syncytia assay)	0.24 µg/mL 0.73 µg/mL 4.26 µg/mL 1.14 µg/mL 23.75 µg/mL 31.86 µg/mL 1.09 µg/mL 0.95 µg/mL 1.12 µg/mL 71.76 µg/mL97.63 µg/ml	**anti-HIV-1**; electrostatic interactions with basic amino acid residues of Tat	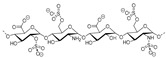	[53]
	fucose containing	Nha Trang bay, Vietnam	*Sargassum mcclurei, Sargassum polycystum,* and *Turbinara* *Ornate* brown seaweeds	U373-CD4-CXCR4 cells 211 infected with pseudotype viral IC_50_—inhibition (F_SP_ crude extract)-(p24 ELISA) IC_50_—inhibition (F_TO_ crude extract)-(p24 ELISA) IC_50_—inhibition (F_SM_ crude extract)-(p24 ELISA)	0.34 µg/mL 0.39 µg/mL 0.96 µg/mL	**anti-HIV-1**; inhibition of the early phase of infection, by blocking the virus attachment and entry into the host cells		[56]
	ascophyllan (**4**)	not disclosed	different sources	IC_50_—inhibition of HIV-1_R9_-real-time PCR	1.3 µg/mL	**anti-HIV-1**; early step of HIV-1 (R9 and JR-Fl) infection; inhibition of VSV-G-pseudotyped HIV-1 infection in HeLa cells	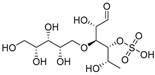	[59]
	fucoidan S (**5**) fucoidan A (**6**)	not disclosed	different sources	IC_50_—inhibition of HIV-1_R9_-real-time PCR (fucoidan S) IC_50_—inhibition of HIV-1_R9_-real-time PCR (fucoidan A)	0.3 µg/mL 0.6 µg/ml	**anti-HIV-1**; early step of HIV-1 (R9 and JR-Fl) infection; inhibition of VSV-G-pseudotyped HIV-1 infection in HeLa cells	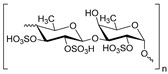	[59]
	chondroitin sulfate (**7**)	not disclosed		EC_50_—HIV-1 p24 detection-PBMC assay-inhibition of HIV-1_IIIB,_ HIV-1_L10R/M46I/L63P/V82T/I84V_, HIV-1_A17_, HIV-1_RF,_ and HIV-1_RF/V82F/184V_ strains	0.01–0.08 μM	**anti-HIV-1**; inhibition of HIV-1 replication; inhibition of the HIV-1 entry	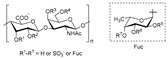	[61]
Lectins	KAA-1	not disclosed	red alga *Kappaphycus alvarezii*	IC_50_—neutralization assay in Jurkat cells (median tissue culture infectious dose (TCID50) method using Jurkat cells)	9.2 nM	**anti-HIV-1**; inhibition of the HIV-1 entry		[87]
	KAA-2	not disclosed	red alga *Kappaphycus alvarezii*	IC_50_—neutralization assay in Jurkat cells (median tissue culture infectious dose (TCID50) method using Jurkat cells)	7.3 nM	**anti-HIV-1**; inhibition of the HIV-1 entry		[87]
Peptides	stellettapeptin A (**8**)	north-western Australia	marine sponge *Stelletta sp*.	EC_50_—inhibition of the cytotoxic effect upon HIV-1 infection	23 nm	**anti-HIV-1**; cytopathic effect of HIV-1 infection	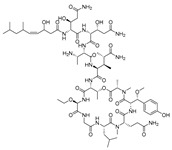	[92]
	stellettapeptin B (**9**)	north-western Australia	marine sponge *Stelletta sp*.	EC_50_—inhibition of the cytotoxic effect upon HIV-1 infection	27 nm	**anti-HIV-1**; cytopathic effect of HIV-1 infection	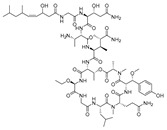	[92]
Bromotyrosine derivatives	aeroplysinin-1 (**10**)	Colombia	marine sponge *Verongula rigida*	% of inhibition of HIV-1 replication by flow cytometry % of reverse transcription inhibition (qPCR analysis of late transcripts) % of nuclear import inhibition (qPCR analysis of 2-LTR transcript) % of HIV entry inhibition (viral infectivity assay)	74% of inhibition at 20 µM 48% of inhibition at 10 µM 67% of inhibition at 10 µM dose dependent manner 2–20%	**anti-HIV-1**; inhibition of HIV-1 replication, RT, nuclear import and entry	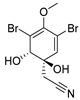	[93]
	3,5-dibromo-*N*,*N*,*N*,*O*-tetramethyl Tyraminium (**13**)	Colombia	marine sponge *Verongula rigida*	% of HIV entry inhibition (viral infectivity assay)	dose depended manner 14–30%	**anti-HIV-1**; inhibition of HIV-1 entry	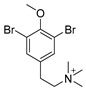	[93]
	19-deoxy fistularin 3 (**15**)	Colombia	marine sponge *Verongula rigida*	% of reverse transcription inhibition (qPCR analysis of early transcripts) % of reverse transcription inhibition (qPCR analysis of late transcripts) % of nuclear import inhibition (qPCR analysis of 2-LTR transcript)	35% inhibition at 20 µM 11% inhibition at 20 µM 62% inhibition at 20 µM	**anti-HIV-1**; inhibition of HIV-1 replication, RT, nuclear import	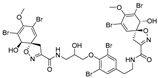	[93]
	purealidin B (**16**)	Colombia	marine sponge *Verongula rigida*	% of inhibition of HIV-1 replication by flow cytometry % of reverse transcription inhibition (qPCR analysis of early transcripts) % of reverse transcription inhibition (qPCR analysis of late transcripts) % of nuclear import inhibition (qPCR analysis of 2-LTR transcript) % of HIV entry inhibition (viral infectivity assay)	57% of inhibition at 80 µM58% of inhibition at 20 µM 34% of inhibition at 20 µM 66% of inhibition at 20 µM dose depended manner 2–11%	**anti-HIV-1**; inhibition of HIV-1 replication, RT, nuclear import and entry	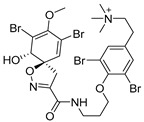	[93]
	fistularin 3 (**17**)	Colombia	marine sponge *Verongula rigida*	% of reverse transcription inhibition (qPCR analysis of late transcripts) % of nuclear import inhibition (qPCR analysis of 2-LTR transcript) % of HIV entry inhibition (viral infectivity assay)	24% of inhibition at 5 µM, 47% of inhibition at 10 µM, dose depended manner 11–13%	**anti-HIV-1**; inhibition of, HIV-1 RT, nuclear import and HIV-1 entry	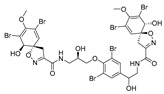	[93]
	3-bromo- 5-hydroxy-O-methyltyrosine (**18**)	Colombia	marine sponge *Aiolochroia crassa*	% of inhibition of HIV-1 replication by flow cytometry % of reverse transcription inhibition (qPCR analysis of early transcripts) % of reverse transcription inhibition (qPCR analysis of late transcripts) % of nuclear import inhibition (qPCR analysis of 2-LTR transcript) % of HIV entry inhibition (viral infectivity assay)	47% of inhibition at 80 µM, 54% of inhibition at 160 µM, 50% of inhibition at 40 µM, 73% of inhibition at 80 µM, dose depended manner 2–12%	**anti-HIV-1**; inhibition of HIV-1 replication, RT, nuclear import and entry	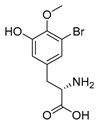	[93]
Peptides	APCHP (**21**)	not disclosed	Alaska pollack	EC_50_—against anti-HIV-1 induced cell lysis (MTT assay) EC_50_—HIV-1-induced RT activation in MT-4 cells EC_50_—against p24 production (western blot)	459 µM (0.403 mg/mL) 374 µM (0.327 mg/mL) 405 µM (**0.356 mg/mL**)	**anti-HIV-1**; inhibition of induced syncytia formation by interference of HIV fusion inhibition of cell lysis, RT activity and production of p24 antigen	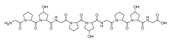	[94]
	SM-peptide	not disclosed	*Spirulina maxima*	IC_50_—protective activity on HIV-1-induced cell lysis-MTT assay % of RT Inhibition in HIV-1-infected cells (reverse transcriptase assay kit) % of HIV-1 p24 antigen production (p24 antigen production assay)	0.691 mM (0.475 mg/mL) 90% inhibition at 1.093 mM (0.75 mg/mL) 95% of inhibition at 1.093 mM (0.75 mg/mL)	**anti-HIV-1**; inhibition of the HIV-1 RT activity and p24 antigen production	Leu-Asp-Ala-Val-Asn-Arg	[95]
Alkaloids	aspernigrin C (**22**)	Yongxing Island, South China Sea Yongxing Island, South China Sea	marine fungus *Aspergillus niger* SCSIO Jcw6F30 isolated from marine alga *Sargassum sp*.	IC_50_—inhibitory effects on infection by CCR5-tropic HIV-1 SF162 in TZM-bl cells	4.7 μM	**anti-HIV-1**	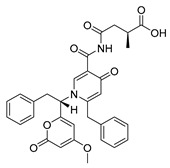	[96]
	malformin C (**23**)	Yongxing Island, South China Sea Yongxing Island, South China Sea	marine fungus *Aspergillus niger* SCSIO Jcw6F30 isolated from marine alga *Sargassum sp*.	IC_50_—inhibitory effects on infection by CCR5-tropic HIV-1 SF162 in TZM-bl cells	1.4 μM	**anti-HIV-1**	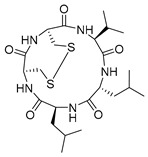	[96]
	eutypellazine E (**24**)	South Atlantic Ocean	deep-sea sediment fungus *Eutypella sp.* MCCC 3A00281	IC_50_—anti-HIV bioassay-pNL4.3.Env-.Luc co-transfected 293T cells	3.2 μM	**anti-HIV-1**; inhibitory effects against HIV-1 replication	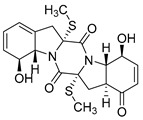	[97]
	eutypellazine J (**25**)	South Atlantic Ocean	deep-sea sediment fungus *Eutypella sp.* MCCC 3A00281	IC_50_—anti-HIV bioassay-pNL4.3.Env-.Luc co-transfected 293T cells reactivation activity-In vitro latent HIV reactivating assay-flow cytometry-based screening	4.9 μM 80 μM	**anti-HIV-1**; inhibitory effects against HIV-1 replication, latency reactivating agent	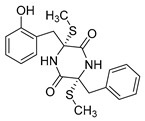	[97]
	debromo- hymenialdisine (**26**)	Coral reefs in the Red Sea	*S. carteri* sponge extract	% of reduction of HIV-1 replication-cell-based assay	30% of inhibition at 13 μM	**anti-HIV 1**; decrease the transcription of the HIV-1, abrogate the G2-checkpoint of the cell cycle	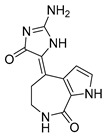	[98]
	Hymenialdisine (**27**)	Coral reefs in the Red Sea	*S. carteri* sponge extract	% of reduction of HIV-1 replication-cell-based assay	<40% of inhibition at 3.1 µM	**anti-HIV 1**; decrease the transcription of the HIV-1, abrogate the G2-checkpoint of the cell cycle	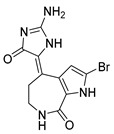	[98]
	Oroidin (**28**)	Coral reefs in the Red Sea	*S. carteri* sponge extract	% of inhibition - HIV-1 RT biochemical assay % of reduction of HIV-1 replication-cell-based assay	90% of inhibition at >25 μM 50% of inhibition at 50 μM	**anti-HIV-1**; inhibition of HIV-1 RT, reduction of HIV-1 replication	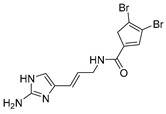	[98]
	3-(phenetyl amino) demethyl(oxy) aaptamine (**29**)	Woody Island (Yongxing, Hainan, China) and Seven Connected Islets in the South China Sea	*A. aptos* sponge extract	% of inhibition against HIV-1 replication-anti-HIV-1 activity assay-cell-based VSVG/HIV-1 pseudotyping system	88% of inhibition at 10 μM	**anti-HIV-1**; inhibitory effects against HIV-1 replication	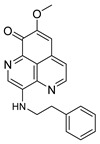	[99]
	3-(isopentyl amino) demethyl(oxy) aaptamine (**30**)	Woody Island (Yongxing, Hainan, China) and Seven Connected Islets in the South China Sea	*A. aptos* sponge extract	% of inhibition against HIV-1 replication-anti-HIV-1 activity assay-cell-based VSVG/HIV-1 pseudotyping system	72.3% of inhibition at 10 μM	**anti-HIV-1**; inhibitory effects against HIV-1 replication	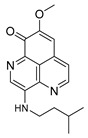	[99]
	bengamide A (**31**)	not disclosed	screening of previously isolated compounds (originally isolated from the sponge *Jaspis* cf. *coriacea*)	EC_50_—multi-cycle viral replication assay % inhibition of p24^Gag^ production-of PBMC assay-p24^Gag^ was quantified by ELISA EC_50_—inhibition of LTR promoter-driven gene expression-LTR-based reporter assays	0.015 μM >90% of inhibition at 0.3 μM 0.17 μM	**anti-HIV-1**; inhibition of NF-κB-mediated retroviral gene expression	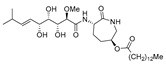	[100]
	haliclony- cyclamine A + B (**32**)	not disclosed	screening of previously isolated compounds	EC_50_—multi-cycle viral replication assay	3.8 μM	**anti-HIV-1**; inhibitory effects against HIV-1 replication	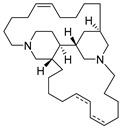	[100]
	keramamine C (**33**)	not disclosed	screening of previously isolated compounds	EC_5_—multi-cycle viral replication assay	3.4 μM	**anti-HIV-1**; inhibitory effects against HIV-1 replication	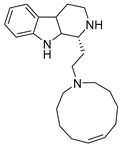	[100]
	stachybotrin D (**34**)	Xisha Island, China	sponge *Xestospongia testudinaris*	EC_50_—inhibitory Effects on Wild-Type and NNRTI-Resistant HIV-1 Replication: EC_50_—inhibition of VSVG/HIV-1_wt_ EC_50_—inhibition of VSVG/HIV-1_RT_-_K103N_ EC_50_—inhibition of VSVG/HIV-1_RT__-__L100I,K103N_ EC_50_—inhibition of VSVG/HIV-1_RT__-__K103N,V108I_ EC_50_—inhibition of VSVG/HIV-1_RT__-__K103N,G190A_ EC_50_—inhibition of VSVG/HIV-1_RT__-__K103N_,_P225H_	8.4 μM 7.0 μM 23.8 μM 13.3 μM 14.2 μM 6.2 μM	**anti-HIV-1**; HIV-1 RT inhibition (inhibitory effects on wild type and five NNRTI-resistant HIV-1 strains)	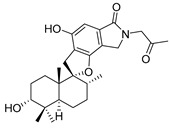	[101]
Diterpenes	dolabelladienol A (**35**)	Atol das Rocas, in Northeast Brazil	brown alga *Dictyota pfaffii*	EC_50_—inhibition of the cytopathic effect of HIV-1-MT-2 cells—MTT method	2.9 μM	**anti-HIV-1**; - inhibition of the cytopathic effect of HIV-1	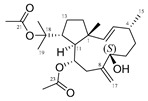	[102]
	dolabelladienol B (**36**)	Atol das Rocas, in Northeast Brazil	brown alga *Dictyota pfaffii*	EC_50_—inhibition of the cytopathic effect of HIV-1-MT-2 cells—MTT method	4.1 μM	**anti-HIV-1**; - inhibition of the cytopathic effect of HIV-1	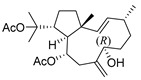	[102]
	dolastane (**38**)	Praia do Velho, Angra dos Reis, in the south of Rio de Janeiro State, Brazil	brown alga *Canistrocarpus cervicornis*	EC_50_—inhibition of HIV-1 replication-CXCR4-tropic HIV-1–MTT method	0.35 μM	**anti-HIV-1**; inhibition of HIV-1 replication, potent effect on HIV-1 infectivity	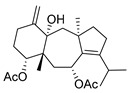	[103]
	dolastane (**39**)	Praia do Velho, Angra dos Reis, in the south of Rio de Janeiro State, Brazil	brown alga *Canistrocarpus cervicornis*	EC_50_—inhibition of HIV-1 replication-CXCR4-tropic HIV-1–MTT method	0.794 μM	**anti-HIV-1**; inhibition of HIV-1 replication, potent effect on HIV-1 infectivity	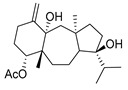	[103]
	secodolastane diterpene (**40**)	Praia do Velho, Angra dos Reis, in the south of Rio de Janeiro State, Brazil	brown alga Canistrocarpus cervicornis	EC_50_—inhibition of HIV-1 replication-CXCR4-tropic HIV-1–MTT method	3.67 μM	**anti-HIV-1**; inhibition of HIV-1 replication	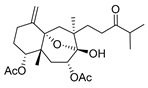	[103]
	8,10,18-trihydroxy-2,6-dolabelladiene (**41**)	Atol das Rocas reef, Brazil	brown alga *Dictyota friabilis*	EC_50_—inhibition of the cytopathic effect of HIV-1-MT-2 cells—MTT method	6.16 μM	**anti-HIV-1**; inhibition of the cytopathic effect of HIV-1	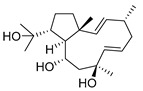	[104,105]
	oxygenated dolabellane (**42**)	Santa Marta Bay (Colombian Caribbean Sea	octocoral *Eunicea laciniata*	EC_50_—inhibition of HIV-1-Inhibition of the cytopathic effect of HIV-1-MT-2 cells—MTT method	3.9 μM	**anti-HIV-1**; inhibition of the cytopathic effect of HIV-1	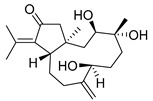	[106]
	oxygenated dolabellane (**43**)	Santa Marta Bay (Colombian Caribbean Sea	octocoral *Eunicea laciniata*	EC_50_—inhibition of the cytopathic effect of HIV-1-MT-2 cells—MTT method	0.73 μM	**anti-HIV-1**; inhibition of the cytopathic effect of HIV-1	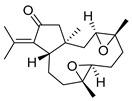	[106]
	oxygenated dolabellane (**44**)	Santa Marta Bay (Colombian Caribbean Sea	octocoral *Eunicea laciniata*	EC_50_—inhibition of HIV-1-Inhibition of the cytopathic effect of HIV-1-MT-2 cells–MTT method	0.69 μM	**anti-HIV-1**; inhibition of the cytopathic effect of HIV-1	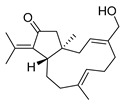	[106]
	8,4′′′-dieckol (**45**)	not disclosed	brown alga, *Ecklonia cava*	Inhibition of syncytia formation on C8166 cells (HIV-1_IIIB_, HIV-1_RF_ and HIV-1_LAI_)-inverted microscope Inhibition of the cytopathic effect of HIV-1-C8166 cells—MTT method	Inhibition in dose-depended manner * Cell viability was more than 90% dose-dependent inhibition	**anti-HIV-1**; inhibition of the cytopathic effects of HIV-1: inhibition of syncytia formation, lytic effects, inhibition of viral p24 antigen production, HIV-1 entry inhibition and RT inhibition	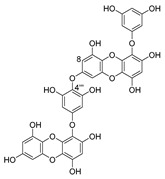	[107]
				Effect on p24 antigen production-p24 antigen capture ELISA and immunoblast analysis RT activity assay—commercial fluorescence RT assay kit	Inhibited 91% activity of HIV-1_IIIB_ RT and approximat ely 80% for rest of the HIV-1 strains tested, HIV-1_RTMDR1_ strain was inhibited at a ratio of 76.1%			
				Inhibition of HIV-1 replication-Luciferase gene reporter assay	At the highest concentration, inhibition was more than 80% for all viral strains except for RTMDR1 (76.33%)			
	penicilli- xanthone A (**46**)	not disclosed	from the jellyfish-derived fungus *Aspergillus fumigates*	IC_50_—inhibition of PXA on infection by CCR5-tropic HIV-1 in TZM-bl cells IC_50_—inhibition of PXA on infection by CXCR4-tropic HIV-1 in TZM-bl cells	0.36 μM 0.26 μM	**anti-HIV-1**; inhibition of infection against CCR5-tropic HIV-1 SF162 and CXCR4-tropic HIV-1 NL4-3	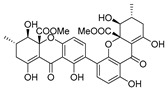	[108]
	docosahexanoic acid (**48**)	not disclosed		In vivo study on male rat models-Male F344 (control) and HIV-1Tg rats		**anti-HIV-1**; neuroprotective effect on neuroinflammations induced by ethanol (in the presence of HIV viral proteins)	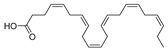	[109]
Phlorotannins and xanthones	radicicol (**49**)	Tutuila, American Samoa	*H. fuscoatra*	EC_50_—In Vitro Model of HIV-1 Latency-high-throughput primary cell-based HIV-1 latency assay	9.1 µM	**anti-HIV-1**; reactivation of latent viral loads in CD4+ T-cells	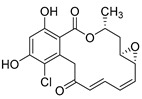	[110]
	pochonin B (**50**)	Tutuila, American Samoa	*H. fuscoatra*	EC_50_—In Vitro Model of HIV-1 Latency-high-throughput primary cell-based HIV-1 latency assay	39.6 µM	**anti-HIV-1**; reactivation of latent viral loads in CD4+ T-cells	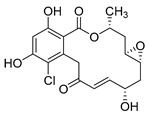	[110]
Auxiliary therapy to HAART therapy—fish oil	pochonin C (**51**)	Tutuila, American Samoa	*H. fuscoatra*	EC_50_—In Vitro Model of HIV-1 Latency-high-throughput primary cell-based HIV-1 latency assay	6.3 µM	**anti-HIV-1**; reactivation of latent viral loads in CD4+ T-cells	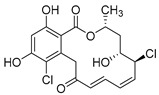	[110]
Others	truncateol O (**52**)	Yongxing Island, Hainan Province of China	sponge-associated fungus *Truncatella angustata*	IC_50_—Anti-HIV bioassays-VSV-G pseudotyped HIV-1–Luciferase assay system	39 µM	**anti-HIV-1**; inhibition of the HIV replication	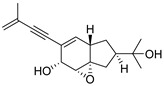	[111]
	truncateol P (**53**)	Yongxing Island, Hainan Province of China	sponge-associated fungus *Truncatella angustata*	IC_50_—Anti-HIV bioassays-VSV-G pseudotyped HIV-1–Luciferase assay system	16.1 µM	**anti-HIV-1**; inhibition of the HIV replication	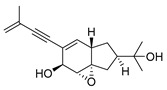	[111]

^a^ Tripeptide conjugates of chitosan (a natural marine byproduct), prepared in the laboratory.
